# Effects of citalopram on blood pressure control in depressive patients with hypertension: A randomized clinical trial

**DOI:** 10.34172/jcvtr.31849

**Published:** 2024-03-13

**Authors:** Hossein Namdar, Elnaz Khani, Sajad Khiali, Naser Safaie, Hedieh Ameli, Gholamreza Rahbari Banaeian, Taher Entezari-Maleki

**Affiliations:** ^1^Cardiovascular Research Center, Tabriz University of Medical Sciences, Tabriz, Iran; ^2^Department of Clinical Pharmacy, Faculty of Pharmacy, Tabriz University of Medical Sciences, Tabriz, Iran; ^3^Department of Pediatrics, Faculty of Medicine, Tabriz Medical Sciences, Islamic Azad University, Tabriz, Iran

**Keywords:** Hypertension, Blood pressure, Depression, Serotonin uptake inhibitors, Citalopram

## Abstract

**Introduction:**

Since there is a bi‐directional interaction between hypertension and depression, we aimed to evaluate the effects of citalopram administration in the management of hypertension.

**Methods:**

A randomized clinical trial was conducted on 72 patients with concomitant depression and hypertension. The intervention group (n=41) received citalopram 20 mg daily plus anti-hypertensive standard treatment, while the control group (n=31) received only the standard treatment. The study’s primary endpoint was in-office blood pressure (BP) measurement at baseline and home BP monitoring in the first and second months after entering the study.

**Results:**

There were no significant differences in baseline systolic BP (163.3±19.6 vs.164.2±20.3 mm Hg; *P*=0.910) and diastolic BP (94.5±13.8 vs. 88.2±14.4; *P*=0.071). After one month, diastolic BP (82.7±11.7 vs. 77.09±12.2; *P*=0.023) was significantly higher in the control group compared to the intervention group. Two months after the intervention, systolic BP (133.8±16.5 vs. 124.5±12.4; *P*=0.009) and diastolic BP (80.7±10.3 vs. 73.7±9.7; *P*=0.002) were significantly decreased in the intervention group compared to the control group.

**Conclusion:**

This study supported the beneficial effects of citalopram in lowering BP in patients with concomitant depression and hypertension.

## Introduction

 Hypertension is a major risk factor for cardiovascular disease (CVD) and results in a higher rate of mortality and morbidity worldwide. Generally, the diagnosis of hypertension has been overlooked in many cases because of lacking specific signs. Of note, the disease remained poorly controlled among diagnosed patients.^1^ According to several studies, most hypertensive patients suffer from depression concomitantly.^2-4^

 Major depression affects approximately 350 million individuals worldwide. It is linked with endothelial dysfunction and arterial stiffening, which may increase the risk of hypertension that can be improved with antidepressant treatment.^5,6^

 Several studies have revealed that depressive patients are more susceptible to develop hypertension. Also, the regulation of blood pressure (BP) is poor in this population.^4,7,8^ Selective Serotonin Reuptake Inhibitors (SSRIs) are commonly used antidepressants that have beneficial cardiovascular effects, such as coronary vasodilation and improvement in endothelial function. ^9,10^ Citalopram is the most selective antidepressant among SSRIs and has small effects on the neuronal reuptake of norepinephrine and dopamine.^11^

 Concerning the epidemiological studies, the possible pathological link between hypertension and depression, and the advantages of combining antidepressant medications with anti-hypertensive drugs, this study evaluated whether antidepressants like SSRI citalopram can control BP effectively.

## Materials and Methods

###  Study design

 The study protocol was confirmed by the Research Ethics Committee of the Tabriz University of Medical Sciences (TBZMED.REC.1397.545) and registered in the Iranian Registry of Clinical Trials (IRCT20111206008307N32). The present study was done in line with the Declaration of Helsinki and the next revisions on ethical principles for clinical studies.^12^ Written informed consent was obtained from all patients or their caregivers. Patients could withdraw from the study at any time.

 This study was a prospective pilot single-blinded, randomized clinical trial (RCT) conducted in the Shahid Madani Heart Center (SMHC), the referral hospital for CVDs in northwest Iran, from September 2018 to January 2019.

###  Study population 

 The inclusion criteria of the study were patients aged between 18 to 80 years with primary hypertension according to the definition of the Eighth Joint National Committee (JNC 8)^13^ and depression symptoms rated with the Hamilton Depression Rating Scale (HAM-D). Patients with kidney or liver failure, patients already on antidepressant medications, a history of white coat syndrome, patients unable to measure their BP, pregnant and lactating women, and patients with secondary hypertension or contraindications to citalopram were excluded.

###  Randomization and study process

 Totally, 72 eligible patients were randomly divided into the intervention (n = 41) and control (n = 31) groups using online GraphPad prism randomization by an independent person not involved in the study.

 In the intervention group, all patients received citalopram 20 mg once daily orally plus standard anti-hypertensive treatment for two months according to the JNC 8 guidelines on the control of BP. In contrast, the control group received only the standard treatment. All of the drugs used in this study are generic with no specific brand. Patients’ demographic data, such as age, sex, body mass index (BMI), laboratory data, drug history, past medical history, and family history of CVD, were noted in data collecting forms.

###  Study endpoints and BP measurements

 The study’s primary endpoint was in-office BP measurement at baseline and home BP monitoring in the first and second months after entering the study.

 An independent nurse not involved in the study process measured all BPs at baseline using an adjusted mercury sphygmomanometer (Rudolf Riester GmbH, Jungingen, Germany) according to the AHA recommendations.^14^ All patients were requested to avoid smoking, eating food, and consuming caffeine or alcohol at least one hour before the measurements. Patients relaxed for five minutes before BP measurements. All evaluations were done in the evening (during clinic activity) three times with 2-minute intervals between them; the three measurements’ mean was considered the main BP. At the first visit, the clinician trained patients to measure and report their BPs in the logbook, which was assessed by the investigators throughout the study period. All patients measured their BPs daily with their digital manometer, validated by the clinic reference mercury manometer (Rudolf Riester GmbH, Jungingen, Germany). As in clinic time, patients were requested to measure their BPs at the evening and not take new drugs. Patients were asked to contact the researchers if they needed to take a new medication, such as corticosteroids, nonsteroidal anti-inflammatory drugs, oral contraceptives, or any medication that might affect BP. The pill-counting technique was used to estimate the compliance of patients with medicine.

###  Statistical analysis

 Data were analyzed in SPSS version 16 (SPSS Inc, Chicago, Illinois, 2007). At first, the Kolmogorov-Smirnov test was performed to evaluate the normality distribution of data. The quantitative data between the groups were compared using Chi-Squared or Fisher’s Exact Test. The repeated measure ANOVA test was used to compare the means of BP during the study time with the Bonferroni adjustment test. The comparison between the two groups was analyzed using the independent t-test. P-values below 0.05 were considered statistically significant.

###  Power and sample size calculation

 The study power was calculated by G-Power (version 3.1.9.2), assuming a type I error probability α = 0.05, n = 72, two groups, and three times serial measurements of BP. The power (1−β error) for systolic BP (SBP) with partial eta-squared (η2) = 0.045 and effect size (F) = 0.001 was calculated 100%. The power (1−β error) for diastolic BP (DBP) with partial eta-squared (η2) = 0.003 and effect size (F) = 0.002 was calculated 100%.

## Results

**Figure 1:**
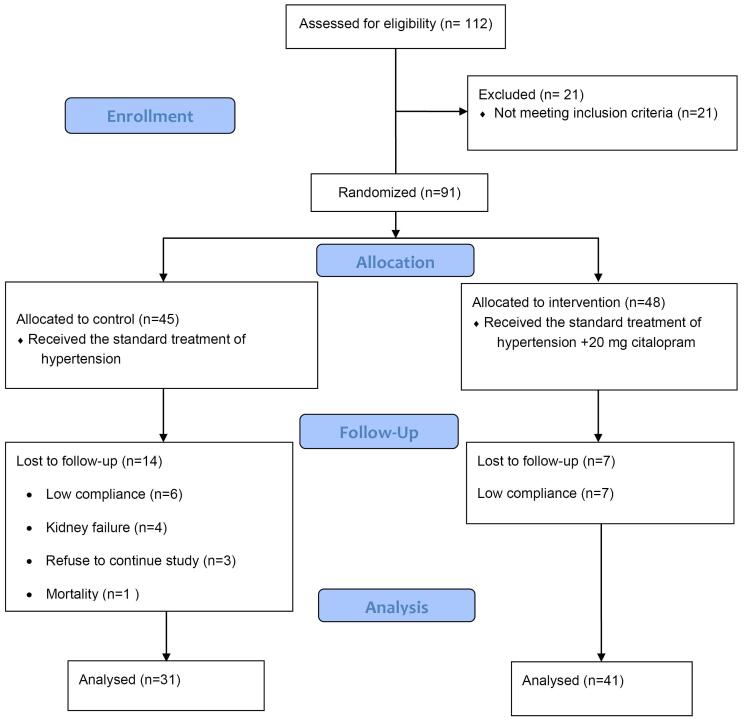
CONSORT flow diagram of the study

 A total of 112 patients were assessed for eligibility. Among these, 91 patients met the inclusion criteria and were randomized to the intervention (n = 46) and control (n = 45) groups. Seven patients in the intervention group and 14 in the control group were lost to follow-up. Finally, 72 patients (41 in the intervention and 31 in the control groups) were analyzed (Figure 1).

 Most patients were female, and the mean age of patients was 55.5 ± 11.6 and 56.6 ± 12.3 in the control and intervention groups, respectively. As shown in Table 1, there were no significant differences regarding the baseline demographic and clinical data of patients between the two groups (*P* > 0.05).

**Table 1:** Baseline demographic and clinical information of the study population

**Demographic/clinical data**	**Intervention** **(n=41)**	**Control** **(n=31)**	***P*** ** value**
Sex, male, n (%)	7 (17)	8 (26)	0.36
Weight (kg), mean ± SD	73.5 ± 8.7	75.0 ± 13.1	0.57
Serum creatinine (mg/dl), mean ± SD	0.92 ± 0.2	0.95 ± 0.2	0.61
Diabetes Mellitus, n (%)	7 (17)	3 (9.6)	0.36
Dyslipidemia, n (%)	9 (21.9)	10 (32.2)	0.70
Other diseases, n (%)	9 (21.9)	10 (32.2)	0.32
Positive family history for ACS, n (%)	32 (78)	20 (64.4)	0.20
Angiotensin Receptor Blocker, n (%)	26 (63)	16 (51)	0.31
Beta-blocker, n (%)	17 (41)	13 (41)	0.96
Calcium Chanel Blocker, n (%)	9 (21.9)	10 (32.2)	0.32
Hydrochloride, n (%)	5 (16.1)	5 (16.1)	0.63
Furosemide, n (%)	2 (4.8)	2 (6.4)	1.00
Aspirin, n (%)	11 (26.8)	4 (12.9)	0.15
HMG-CoA reductase inhibitors, n (%)	9 (21.9)	8 (25.8)	0.70
Metformin, n (%)	6 (14.6)	2 (6.4)	0.27
Sulfonylureas, n (%)	3 (7.31)	5 (16.1)	0.28
Other drugs, n (%)	5 (12.1)	9 (29)	0.07
Smoking, n (%)	5 (12.1)	2 (6.4)	0.70
Receiving one anti-hypertensive drug, n (%)	18 (43.9)	11 (35.4)	0.47
Receiving two anti-hypertensive drugs, n (%)	7 (17)	9 (29)	0.22
Receiving three anti-hypertensive drugs, n (%)	3 (7.3)	2 (6.4)	1.00
Severe depression, n (%)	7 (17)	10 (32.2)	0.13
Very severe depression, n (%)	24 (58.5)	21 (67.7)	0.13

SD, standard deviation; ACS, acute coronary syndrome; HMG-CoA, 3-hydroxy-3 methylglutaryl–coenzyme A

###  BP changes

**Figure 2:**
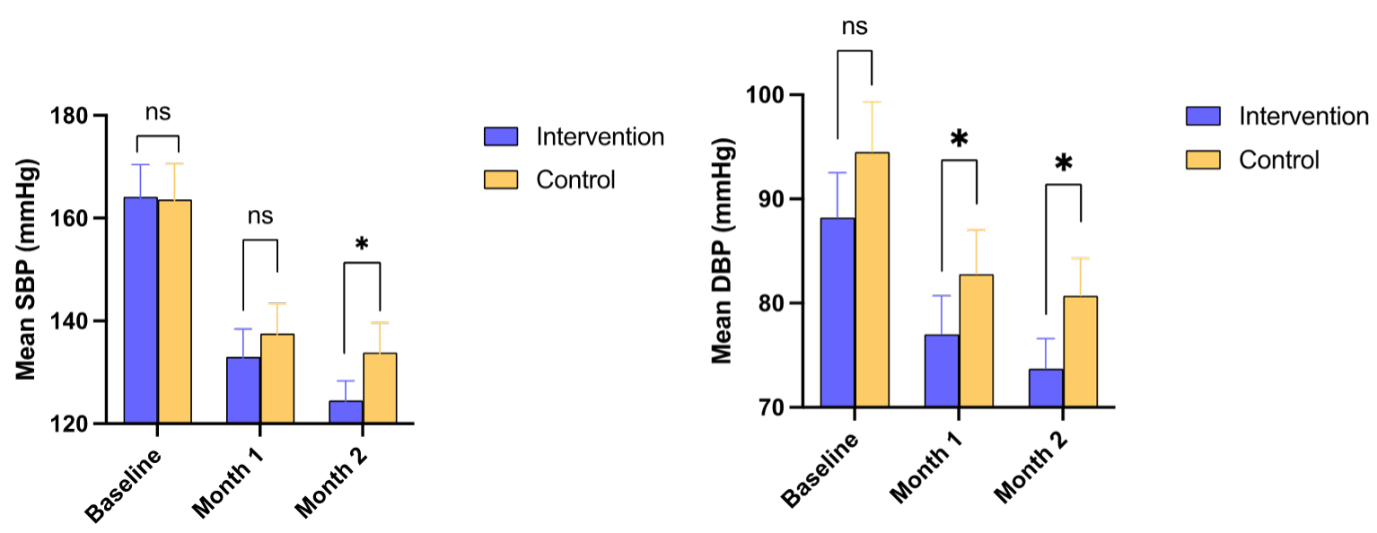
The mean systolic blood pressure (SBP) and diastolic blood pressure (DBP) during the study period

 As shown in Table 2, baseline SBP and DBP were comparable between the two groups. Similarly, no significant difference was documented in month 1 SBP (133.0 ± 17.6 vs. 137.5 ± 16.5 mmHg, *P* = 0.275); however, month 1 DBP (77.09 ± 12.2 vs. 82.7 ± 11.7 mmHg; *P* = 0.023), month 2 SBP (124.5 ± 12.4 vs. 133.8 ± 16.5 mmHg; *P* = 0.009), and month 2 DBP (73.7 ± 9.7 vs. 80.7 ± 10.3 mmHg; *P* = 0.002) were significantly lower in the intervention group compared with the control group (Figure 2).

**Table 2:** The mean blood pressure and HAM-D score at baseline, 1 and 2 months after the study

	**Intervention** **(n=41)**	**Control** **(n=31)**	***P*** ** value**
**Blood pressure changes, mmHg, (mean±SD)**			
SBP (Baseline)	164.2 ± 20.3	163.6 ± 19.6	0.91
SBP (Month 1)	133 ± 17.6	137.5 ± 16.5	0.27
SBP (Month 2)	124.5 ± 12.4	133.8 ± 16.5	0.00
DBP (Baseline)	88.2 ± 14.1	94.5 ± 13.8	0.07
DBP (Month 1)	77.0 ± 12.2	82.8 ± 11.7	0.02
DBP (Month 2)	73.7 ± 9.7	80.7 ± 10.3	0.00
**HAM-D score changes (mean±SD)**			
Baseline (very severe depression)	28.2 ± 3.0	26.8 ± 2.7	0.08
Month 2 (very severe depression)	17.5 ± 3.9	23.5 ± 3.6	0.001
Baseline (severe depression)	21.3 ± 1.1	20.4 ± 1.1	0.13
Month 2 (severe depression)	12.0 ± 4.0	18.2 ± 1.3	0.006

SD, standard deviation; SBP, systolic blood pressure; DBP, diastolic blood pressure; HAM-D, Hamilton Depression Rating Scale. *P* value< 0.05 statistically significant.

###  Depression scale changes 

 At the baseline, the mean HAM-D score of the patients was similar between the two groups. However, two months after allocation, the mean HAM-D score was significantly lower in the intervention group than the control group with very severe (17.5 ± 3.9 vs. 23.5 ± 3.6; *P* = 0.001) and severe depression (12.0 ± 4.0 vs. 18.2 ± 1.3; *P* = 0.006) (Table 2).

## Discussion

 To our knowledge, this may be the first RCT in our region that assessed the effect of citalopram on BP in hypertensive patients with depression. This study showed that adding citalopram to anti-hypertensive medications significantly decreased SBP and DBP after two months.

 Since the prevalence of depression is higher among hypertensive patients and vice versa, epidemiological studies suggested that there may be a relationship between the two diseases.^4^ A meta-analysis of cohort studies including 22367 patients revealed that depression increased the risk of hypertension during 9.6 years (adjusted relative risk 1.42; *P* = 0.009).^15^

 Based on the results of a prospective study, depressive patients who responded to citalopram and risperidone had significantly improved SBP and DBP.^6^

 A multicenter, placebo-controlled RCT conducted by Glassman et al evaluated the effect of sertraline on left ventricular ejection fraction in 369 depressive patients hospitalized for acute MI or unstable angina. As a secondary variable, the comparison of BP after 24 weeks was reported to be similar between the two groups. However, this study did not categorize the patients based on the severity of depression. Also, patients with uncontrolled hypertension did not enter this study.^16^

 According to the study by Peixoto et al., it has been shown that, in patients with hypertension and depression, 10 mg of escitalopram did not affect SBP and DBP significantly after 8 weeks. Patients in this study had stage 1 or stage 2 hypertension. All patients (15 in the escitalopram group and 15 in the placebo group) received 50 mg of losartan plus 12.5 mg of hydrochlorothiazide. However, based on the Seventh Joint National Committee statement (JNC 7), the treatment protocols for stage 1 and stage 2 hypertension are different. Also, the sample size of this study was small, and the power was not defined.^17^

 Another RCT assessed the role of antidepressant medication on BP in 70 elderly patients with hypertension and depression. Of them, 35 patients received 20 mg of citalopram daily, and all patients under study received 5 mg of amlodipine daily. After three months, SBP and DBP were significantly improved in the citalopram group compared to the control group. However, the results cannot be extended to patients under 60 years.^18^

 The current study has several strong points in assessing citalopram’s effect on hypertension. First, all BP measurements were based on AHA guidelines, while in most of the previous studies, the method of BP measurement was not indicated. Second, this study directly evaluated the role of citalopram on hypertension and supported the beneficial effect of that on the BP management of patients with depression. Thus, it leads clinicians to be aware of depressive signs in hypertensive patients and consider antidepressant drugs for better management of hypertension.

 Although ambulatory BP monitoring is considered the gold standard for hypertension diagnosis and BP control, home BP monitoring is a reliable alternative with more feasibility, greater patient acceptance, and lower cost.^19^ Hence, self-measured BPs on subsequent visits could accurately represent patients’ BP.

 The current article might have some limitations. Due to time and cost limitations, this study was not controlled with a placebo and was conducted only in a single center for a limited follow-up period. Second, only the primary endpoint of this study was underpowered. As another limitation of our study, participants’ dietary regimen is not necessarily similar.

## Conclusion

 The results of this study revealed that in comparison with the control group, adding citalopram to anti-hypertensive medications significantly improved BP levels and depression scores in hypertensive patients with depression after two months. However, large-scale studies are required to confirm these findings.

## Competing Interests

 The authors declare no conflicts of interest.

## Ethical Approval

 The study protocol was approved by the Research Ethics Committee of the Tabriz University of Medical Sciences (TBZMED.REC.1397.545), and written informed consent was obtained from all patients.

## Funding

 This research did not receive any specific grant from funding agencies.
